# Heterologous Array Analysis in Pinaceae: Hybridization of *Pinus Taeda* cDNA Arrays With cDNA From Needles and Embryogenic Cultures of *P. Taeda*, *P. Sylvestris* or *Picea Abies*

**DOI:** 10.1002/cfg.199

**Published:** 2002-08

**Authors:** Leonel van Zyl, Sara von Arnold, Peter Bozhkov, Yongzhong Chen, Ulrika Egertsdotter, John MacKay, Ronald R. Sederoff, Jing Shen, Lyubov Zelena, David H. Clapham

**Affiliations:** 1 Forest Biotechnology Group Centennial Campus, Box 7247 North Carolina State University Raleigh NC 27695 USA; 2 Department of Forest Genetics Swedish University of Agricultural Sciences Box 7027 Uppsala S-750 07 Sweden; 3 Institute of Paper Science and Technology Forest Biology Group 500 10th Street NW Atlanta GA 30318 USA; 4 Bioinformatics Research Center Centennial Campus North Carolina State University Raleigh NC 27695 USA

## Abstract

Hybridization of labelled cDNA from various cell types with high-density arrays of expressed sequence tags is a powerful technique for investigating gene expression. Few conifer cDNA libraries have been sequenced. Because of the high level of sequence conservation between *Pinus* and *Picea* we have investigated the use of arrays from one genus for studies of gene expression in the other. The partial cDNAs from 384 identifiable genes expressed in differentiating xylem of *Pinus taeda* were printed on nylon membranes in randomized replicates. These were hybridized with labelled cDNA from needles or embryogenic cultures of *Pinus taeda*, P. *sylvestris* and *Picea abies*, and with labelled cDNA from leaves of *Nicotiana tabacum*. The Spearman correlation of gene expression for pairs of conifer species was high for needles (r^2^ = 0.78 − 0.86), and somewhat lower for embryogenic cultures (r^2^ = 0.68 − 0.83). The correlation of gene expression for tobacco leaves and needles of each of the three conifer species was lower but sufficiently high (r^2^ = 0.52 − 0.63) to suggest that many partial gene sequences are conserved in angiosperms and gymnosperms. Heterologous probing was further used to identify tissue-specific gene expression over species boundaries. To evaluate the significance of differences in gene expression, conventional parametric tests were compared with permutation tests after four methods of normalization. Permutation tests after Z-normalization provide the highest degree of discrimination but may enhance the probability of type I errors. It is concluded that arrays of cDNA from loblolly pine are useful for studies of gene expression in other pines or spruces.


**Comparative and Functional Genomics**
*Comp Funct Genom* 2002; **3**: 306–318.
Published online in Wiley InterScience (www.interscience.wiley.com). **DOI:** 10.1002/cfg.199

**Research Article**

### Heterologous array analysis in Pinaceae: hybridization of *Pinus taeda* cDNA arrays with cDNA from needles and embryogenic cultures of *P. taeda*, *P. sylvestris* or *Picea abies*

Leonel van Zyl,[1] Sara von Arnold,[2] Peter Bozhkov,[2] Yongzhong Chen,[2] Ulrika Egertsdotter,[3] John MacKay,[3] Ronald R. Sederoff,[1] Jing Shen,[4] Lyubov Zelena[2] and David H. Clapham[2]*

[1] *Forest Biotechnology Group, Centennial Campus, Box 7247, North Carolina State University, Raleigh, NC 27695, USA*
[2] *Department of Forest Genetics, Swedish University of Agricultural Sciences, Box 7027, S-750 07 Uppsala, Sweden*
[3] *Institute of Paper Science and Technology, Forest Biology Group, 500 10th Street NW, Atlanta, GA 30318, USA*
[4] *Bioinformatics Research Center, Centennial Campus, North Carolina State University, Raleigh, NC 27695, USA*

**Correspondence to:*
*David H. Clapham, Dept. of Forest Genetics, Swedish University of Agricultural Sciences, Box 7027, S0750 07, Uppsala, Sweden.*
*E-mail:*
*David.Clapham@sgen.slu.se*

Received: 4 March 2002
Accepted: 14 June 2002

### Abstract

**Hybridization of labelled cDNA from various cell types with high-density arrays of expressed sequence tags is a powerful technique for investigating gene expression. Few conifer cDNA libraries have been sequenced. Because of the high level of sequence conservation between *Pinus* and *Picea* we have investigated the use of arrays from one genus for studies of gene expression in the other. The partial cDNAs from 384 identifiable genes expressed in differentiating xylem of *Pinus taeda* were printed on nylon membranes in randomized replicates. These were hybridized with labelled cDNA from needles or embryogenic cultures of *Pinus taeda*, *P. sylvestris* and *Picea abies*, and with labelled cDNA from leaves of *Nicotiana tabacum*. The Spearman correlation of gene expression for pairs of conifer species was high for needles ($r^2 = 0.78 - 0.86$), and somewhat lower for embryogenic cultures ($r^2 = 0.68 - 0.83$). The correlation of gene expression for tobacco leaves and needles of each of the three conifer species was lower but sufficiently high ($r^2 = 0.52 - 0.63$) to suggest that many partial gene sequences are conserved in angiosperms and gymnosperms. Heterologous probing was further used to identify tissue-specific gene expression over species boundaries. To evaluate the significance of differences in gene expression, conventional parametric tests were compared with permutation tests after four methods of normalization. Permutation tests after Z-normalization provide the highest degree of discrimination but may enhance the probability of type I errors. It is concluded that arrays of cDNA from loblolly pine are useful for studies of gene expression in other pines or spruces. Copyright © 2002 John Wiley & Sons, Ltd.**

**Keywords:** cDNA array; conifer; normalization; functional genomics; pine; spruce

### Introduction

Approaches to studying the genetics and physiology of conifers and other forest trees are being radically altered by the arrival of 'high-throughput' methods. Large-scale DNA sequencing (e.g. Allona *et al.*, 1998) was followed by the introduction of methods to enable large-scale analysis of function, such as the use of high-density arrays of hundreds or thousands of cDNAs printed on glass (Shena *et al.*, 1995) or membranes (e.g. Heller *et al.*, 1997; Richmond *et al.*, 1999; Cairney *et al.*, 1999). These arrays are hybridized with suitably labelled cDNA derived from the material of interest. The intensity of the label over each spot provides an estimate of steady-state mRNA relative abundance for large numbers of genes, potentially all the genes active in the tissue. For a recent review of the methodology

Copyright © 2002 John Wiley & Sons, Ltd.

for microarrays on glass, see Hegde *et al.* (2000). Arrays on membranes and on glass give comparable results, (Richmond *et al.*, 1999), but arrays on glass are preferred when large numbers of genes are being surveyed.

DNA microarrays have already found wide use for plants. Studies of *Arabidopsis* were included in the earliest report (Schena *et al.*, 1995); and more recent reports include those of Ruan *et al.* (1998), Schenk *et al.* (2000), Girke *et al.* (2000) and Maleck *et al.* (2000). For woody species there is a study of genes regulating somatic embryogenesis in *Pinus taeda* (Cairney *et al.*, 1999), of wood formation in *Pinus taeda* (Whetten *et al.*, 2001) and of wood formation in *Populus* (Hertzberg *et al.*, 2001a,b).

Microarray analysis will be more widely applicable if arrays constructed from *Pinus taeda* DNA, currently the only conifer for which extensive sequence information is publicly available, can be used for detection of gene activity in other conifer species. Recently a microarray of 2600 *Arabidopsis* genes expressed in seeds was hybridized with labelled cDNA from *Arabidopsis* or from another cruciferous species, *Brassica napus*; only a minor loss of sensitivity was noted with the heterologous probe (Girke *et al.*, 2000). Presumably the stringency of hybridization does not resolve most of the differences in sequence between these species. The present study compares results obtained when arrays of *Pinus taeda* cDNA, printed on membranes, are hybridized with labelled cDNA from the same species, from another species of the same genus (*P. sylvestris*), from another genus of the family Pinaceae or from an angiosperm (*Nicotiana tabacum*).

### Materials and methods

### Plant material

The array was printed with clones of a cDNA library constructed from RNA isolated from the differentiating xylem of compression or side wood of three 6 year-old *Pinus taeda* (loblolly pine) trees (Allona *et al.*, 1998), from shoot-tips 2 cm from the apex, or from immature pollen cones. The arrays were hybridized with labelled cDNA derived from:

1. Needles of seedlings of *P. taeda* raised in growth cabinets for 7 weeks in continuous light from fluorescent tubes supplemented with tungsten filament lamps at about 200 μmol/m$^2$/s at 25 °C. The seeds were treated with 1% hydrogen peroxide under fluorescent light, 100 μmol/m$^2$/s, with two changes over 3 days, for disinfection and to induce germination before planting in pots containing a grit : sand : vermiculite : perlite mixture (4 : 2 : 2 : 8). From the cotyledonary stage the seedlings were watered with a dilute nutrient solution (Ingestad, 1979).
2. Needles of seedlings of *Pinus sylvestris* (Scots pine) raised as above except that after 4 weeks at 25 °C the seedlings were transferred to 20 °C for a further 2 weeks before harvesting. The seeds, from a seed orchard at Hultsfred, central Sweden, lot S21 A8210001, were soaked in water overnight before planting.
3. Needles of seedlings of *Picea abies* raised as for *Pinus sylvestris*. The seeds were from a seed orchard at Saleby, central Sweden.
4. An embryogenic culture of *P. taeda* line 344 maintained on a proprietary medium at the Institute of Paper Science and Technology, Atlanta, GA, USA.
5. An embryogenic culture of *P. sylvestris*, line F2, maintained on DCR medium (Gupta and Durzan, 1986).
6. An embryogenic culture of *P. abies* line 95 : 88 : 17 maintained on LP proliferation medium (Bozhkov and von Arnold, 1998).
7. Leaves of *Nicotiana tabacum* cv. *xanthi*, raised in compost in the greenhouse. In short, material consisted of needles from conifer seedlings, proliferating somatic embryogenic cultures of conifer species, or tobacco leaves.

### Genes in the array

Genes from the cDNA libraries were selected to represent various functional categories, together with 10 negative control genes from organisms phylogenetically remote from plants (Table 1). There is an emphasis on genes related to wood formation, although all categories are represented, including unidentified genes. The putative negative control cDNAs were: the mammalian transcription factors Sp1, Sp2, Sp3 and Sp4; Bt toxin gene from *Bacillus thuringiensis*; the *BAR* gene from *Streptomyces hygroscopicus*; *GFP* (green fluorescent protein) gene from the jellyfish *Aequoria victoria*; a

Copyright © 2002 John Wiley & Sons, Ltd. 

Table 1. Description of genes and number of genes in each class in the array

| Description | Number |
|---|---|
| Phenylpropanoid pathway and related metabolism | 37 |
| Phenylpropanoid pathway | 20 |
| Shikimate pathway, sesquiterpene synthesis | 2 |
| Lignan reductase, glycine hydroxymethyltransferase, flavanol glycosyltransferase | 8 |
| S-adenosylmethionine synthase and carboxylase | 7 |
| Cell wall-related | 59 |
| Laccase | 8 |
| Arabinogalactans, other cell wall proteins | 17 |
| Sucrose and sucrose phosphate synthases, UDP glucose-6-DH, inositol 1-phosphate synthase | 8 |
| Xyloglucan endotransglycosylase, | 6 |
| Cellulose synthase, cellulase | 4 |
| Pectinesterase | 6 |
| Tubulin, actin, anaphase-promoting complex | 10 |
| Stress response, signal transduction, membrane transport proteins | 117 |
| Chaperonins, heat-shock proteins, dnaJ proteins | 15 |
| Glutathione-related, superoxide dismutase, methionine sulphoxide reductase | 13 |
| Peroxidase, peroxiredoxin, catalase, thioredoxin-h | 10 |
| Drought-, cold- and salt-induced proteins, responses to ABA or ethylene | 17 |
| Porins, other membrane transport proteins, lipid transport protein | 26 |
| GTP-binding proteins, cAMP response element binding protein | 8 |
| Calmodulin, protein kinase, protein phosphatase | 15 |
| Peptidyl-prolyl isomerase, cysteine proteinase | 8 |
| Miscellaneous: ADP-ribosylation factor, chitinase, serine carboxypeptidase, spalt1 | 5 |
| Proteasome-associated | 14 |
| Ubiquitin | 4 |
| Ubiqutin-conjugating enzyme | 10 |
| Primary metabolism and information transfer | 62 |
| Respiration, lipid metabolism | 18 |
| Photosynthesis, photorespiration, other chloroplast-related functions | 13 |
| Amino-acid synthesis | 4 |
| Histones, RNA polymerase, DNA-binding proteins, transcription factors | 18 |
| Ribosomal proteins, rRNA, t-RNA synthase, protein synthesis initiation and elongation factors | 7 |
| Negative control genes from unrelated organisms, open control | 11 |
| Genes of unidentified function | 86 |

human globin gene; *gusA* gene from *E. coli*; and the *HPH* (hygromycin phosphotransferase) gene from *E. coli*. Identity of clones was verified by resequencing a sample of 75% of the total after PCR amplification.

### Printing of arrays on membranes

Inserts from plasmid DNA from selected clones of the *P. taeda* cDNA library were amplified by PCR using plasmid-specific primers. Aliquots were run out on 1% agarose electrophoresis gels to verify amplification of a single product. Clones showing multiple bands were replaced with alternative cDNAs. DNA from each of 384 clones (750–1500 ng) in 6 µl was transferred to the wells of each of three 384-well microtitre plates, in separate randomized arrangements. Each microtitre plate was therefore one randomized block, with 384 clones per block, and there were three replicates per clone. To each well was added 3 µl NaOH (0.83 M), the plate was sealed with aluminium foil, and held at 65 °C for 1 h to denature the DNA. The plate was cooled to room temperature and 15 µl neutralizing solution (95% 20× SSPE, 5% gel-loading buffer containing bromophenol blue) added to each well. The cDNA clones were then printed on 15 × 10 cm strips of Hybond $N^+$ nylon membrane using a pin blotter (cat. No VP386, V&P Scientific Inc, San Diego, CA) to give about 3–6 ng

Copyright © 2002 John Wiley & Sons, Ltd. 

DNA per spot. The DNA was cross-linked to the membrane at 120 000 mJ/cm$^2$ using a Biorad UV linker.

### Isolation of RNA and synthesis and labelling of cDNA

The cDNA library from differentiating xylem of *P. taeda* was constructed as described previously (Allona *et al.*, 1998). Total RNA from conifer seedlings was isolated according to Chang *et al.* (1993), from conifer embryogenic cultures using Qiagen RNeasy Plant Mini Kit columns according to the manufacturer's instructions except that the extraction buffer was that of Chang *et al.* (1993). Total RNA was extracted from *Nicotiana* leaves as described by Logemann *et al.* (1987). DNA was removed by treatment with DNAse I (Sigma kit). For first strand cDNA synthesis, 5 µg denatured total RNA was added to a reaction mixture of final volume 20 µl containing 50 mM Tris–HCl, pH 8.3, 75 mM KCl, 3 mM $MgCl_2$, 10 mM DTT, 0.5 mM each dATP, dCTP, dGTP and dTTP, 0.5 µg 17-mer oligo-dT primer mixture with A, C or G at the 5′-prime end to minimize the transcription of long poly-A tails, 40 units RNase Out RNase inhibitor (Life Technologies) and 200 units Superscript II reverse transcriptase (Life Technologies). The mixture was incubated for 1 h at 42 °C and the reaction was terminated by heating to 70 °C for 15 min and cooling on ice. The first strand reaction mixture was then added to a reaction mixture for second strand cDNA synthesis containing second-strand buffer (Life Technologies) according to the manufacturer's instructions and incubated for 2 h at 16 °C. The cDNA was then precipitated with 0.3 M sodium acetate and 2 vol ethanol, centrifuged down, washed in 70% alcohol, redissolved in 34 µl Tris–EDTA (10 mM–1 mM) buffer, pH 8.0, and denatured for 5 min at 95 °C. The cDNA was labelled with fluorescein-11-dUTP by random priming and Klenow fragment in a reaction volume of 50 µl (ECF random prime labelling system, RPN 5751, Amersham Pharmacia).

### Hybridization of the membranes with labelled target and detection and quantification of label

Membranes were prehybridized for 30 min at 60 °C in 15–20 ml 5× SSC, 0.1% SDS, 20-fold dilution of liquid block (Amersham Pharmacia RPN 3601), 5% dextran sulphate (mol weight 500 000) in hybridization bottles (Hybaid). Then labelled target was added to the prehybridization buffer (0.3–1.5 µl/ml buffer) and membranes were hybridized overnight at 60 °C. Membranes were washed for 15 min in preheated 1× SSC, 0.1% SDS at 60 °C and for 10 min in 0.5× SSC, 0.1% SDS at 60 °C. They were then briefly rinsed in freshly made or autoclaved 100 mM Tris–HCl, 300 mM NaCl, pH 7.5 (buffer A) and incubated in the blocking solutions of the kit (RPN 3601) according to the manufacturer's instructions. Membranes were then incubated with anti-fluorescein antibodies coupled to alkaline phosphatase (Roche) diluted 50 000-fold in detection buffer (0.1 M Tris–HCl, 0.1 M NaCl, pH 9.5) for 1 h at room temperature with shaking, using 0.2 ml/cm$^2$ membrane. Membranes were washed for 3 × 10 min in 0.3% Tween 20 in buffer A at room temperature. Then wash buffer was drained and 2 ml CDP-*Star* (Roche) diluted 1 : 5 in detection buffer, was pipetted on to each membrane (15 × 10 cm) for 1.5 min in a room with low lighting. Excess reagent was drained off, the blots wrapped in plastic and exposed for 0.5–60 min to Hyperfilm ECL (Amersham Pharmacia) in a cassette. After development (Figure 1), the autoradiographs were scanned and the images quantified with the Quantity One Image Analysis Program (Biorad Ltd). Local values for background were estimated using the program after initial testing with global values.

### Normalization of data

For each replicate, the pixel values (intensities, estimates of gene expression) for each gene were adjusted so that the lowest scoring gene was zero. Then the data from the various replicates were normalized by each of the following procedures:

- *Normalization by mean*: the intensity for each gene in a particular replicate was divided by the mean intensity for all genes in the replicate and multiplied by 100 so that values were expressed on a scale where 100 was the mean intensity.
- *Student normalization* (Richmond and Somerville, 2000): the intensity of each gene in a replicate was divided by the standard deviation of the intensities of all the genes in the replicate.

Copyright © 2002 John Wiley & Sons, Ltd. 

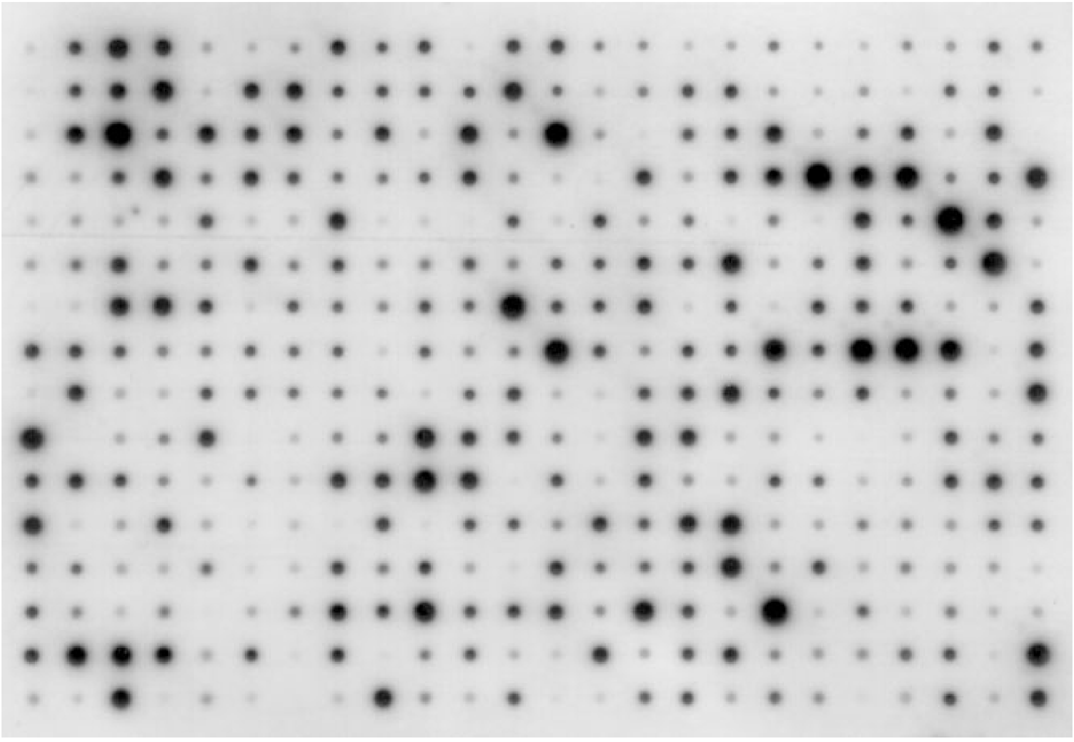

**Figure 1.** Autoradiograph of the chemiluminescent image formed after hybridizing an array of *Pinus taeda* partial cDNAs with labelled cDNA from needles of *Picea abies*

- *Z-score normalization* (Richmond and Somerville, 2000): (a) from the intensity ($\chi_n$) of the $n$th gene in the replicate was subtracted the mean value ($\mu$) for all genes in the replicate, and this difference was divided by the standard deviation ($\sigma$) for the intensities of all the genes in the replicate, i.e. $Z_n = (\chi_n - \mu)/\sigma$; (b) Z-score normalization as above, except that the data were first logarithmically transformed to base 2 before calculating the Z-score. Before taking logs, 1 was added to each gene intensity to avoid problems with the logarithms of small numbers, i.e. $Z_n = [\log_2(\chi_n + 1) - \mu]/\sigma$.
- *Regression normalization* (after Hegde *et al.*, 2000): a linear regression line was fitted to relate the intensities of all the genes in the second replicate to the intensities of the first replicate array of the series under comparison. The intensities of each gene in the second replicate were normalized (rescaled) by dividing by the slope of the regression line; the intensities of the genes in the third and subsequent replicate arrays were then similarly rescaled to those of the first replicate. Where different species or cell-types were being compared, the intensities of each array were related to the first replicate of the first species or cell-type. The principle here is that for closely related samples, many of the genes should be expressed at nearly constant levels. Consequently, a scatterplot of the measured intensities in the $n$th array vs. those in the first array should have a slope of one (Hegde *et al.*, 2000).

### Frequency distribution of the intensity of gene expression

The intensities of each gene in a replicate array were normalized by method 1 (see above) and mean values for each gene over the three replicates were calculated. For one set of calculations the mean values for gene expression were transformed to logs to base 2. The proportion of genes whose intensity fell into various intervals ('bins'), such that 16–19 bins covered the range of expression, was graphed to show the frequency distribution of estimated gene expression.

Copyright © 2002 John Wiley & Sons, Ltd. 

### Testing the significance of changes in gene expression

The significance of apparent changes in gene expression from embryogenic callus to needles that are consistent for all three conifer species was tested by various methods to assess the sensitivity and robustness of the system. Data were normalized by methods 1, 3a, 3b or 4. After applying methods 1, 3a or 4, the data were transformed to log base 2; after method 3a, four (the smallest suitable integer) was added to the normalized values before log transformation to avoid logarithms of negative numbers. The significance of the difference in mean expression for each gene was assessed by $t$-tests. In addition, a permutation method, essentially as described in Good (1993), was applied as follows:

(a) The data were normalized according to method 3b.
(b) A $t$-value, $t_{obs}$, was calculated for each gene for the difference in means between the nine observations for embryogenic callus and the nine observations for needles. Variances of the two groups were assumed unequal (Satterthwaite method).
(c) The labels were rearranged (permuted) between the two groups in all 48 620 possible arrangements, or in a random sample of 1000 arrangements.
(d) The permuted $t$-value, $t^*$, was calculated for each arrangement.
(e) A $p$ value was calculated for each gene as the proportion of $t^*$ values greater than $t_{obs}$.

### Results

### Frequency distribution of gene expression

Since many statistical tests are valid only if the data approximate to a Gaussian distribution, the frequency distribution of expression intensities for all the genes in the array was examined. For untransformed gene expression data normalized by method 1, the frequency distribution was skewed such that the median value was 75–90% of the mean value (Figure 2A and data not shown). This was corrected by the log to base 2 transformation (Figure 2B) after which the frequency distributions did not differ significantly from Gaussian by the Kolmogorov–Smirnov test (data not shown).

### Negative controls

The intensities of the genes were normalized by method 1 and then an average for each gene for the three replicates of each species and both cell-types was calculated. The values were arranged in ascending order of magnitude (data not shown). The first five genes were globin, *Sp4*, *Sp1*, an open control and *BAR*, i.e. four of the 10 putative negative controls were excellent as negative controls, showing only a low background similar to the blank and less than any of the pine genes. Their intensities were all close to 20% of the average gene intensity. A group of negative control genes showing somewhat more hybridization consisted of GFP, Sp2, HPH, the BT toxin and Sp3, respectively in places 21, 30, 37, 48 and

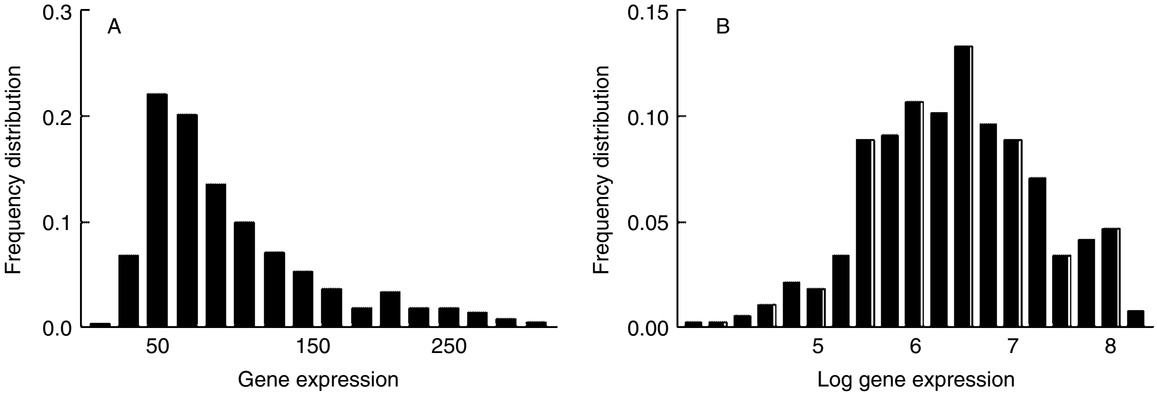

**Figure 2.** Frequency distribution of the mean expression of genes from needles of *Picea abies* estimated after heterologous hybridization to the *Pinus taeda* array. Data were normalized according to method 1 (see Materials and methods) and left untransformed (A) or logarithmically transformed to base 2 (B)

Copyright © 2002 John Wiley & Sons, Ltd. 

51. The intensities were 38–48% of the average gene intensity. The tenth gene, *gusA*, encoding $\beta$-glucuronidase, showed considerable hybridization, occupying place 133 with an intensity of 70% of the average gene. The four most highly expressed (conifer) genes, of intensity 300–330% of the average gene, included one related to a zinc-finger protein, Spalt, one coding for a ribosomal protein, and two of unknown function.

### Pairwise comparisons of gene expression

To test the potential for hybridizing labelled cDNA from one conifer species with arrays of cDNA from another conifer species, the intensities of gene expression were compared after heterologous and homologous hybridization with the *Pinus taeda* array (Table 2). For each comparison, the Pearson correlation coefficients on log-transformed data showed good agreement with, but were slightly higher than, the Spearman (non-parametric) correlation coefficients. The Spearman correlation coefficients were high, 0.88–0.93, for needle comparisons, and nearly as high, 0.83–0.91 for the embryogenic comparisons. For comparisons of different cell-types (embryogenic callus and needles) within the same species, the correlation coefficients were more variable (0.68–0.85). The correlations are graphed for two examples with *P. abies* material in Figure 3.

Some of the calculations were repeated for the subset of 86 unidentified genes, to reduce possible bias towards genes of highly conserved sequence. For needles, the Spearman correlations were slightly higher for this subset than for the complete set, range 0.89–0.94. For embryogenic cultures, the Spearman correlations were lower, range 0.67–0.86.

To test phylogenetically more remote comparisons, labelled cDNA from tobacco leaves was hybridized with the *P. taeda* arrays. The Spearman correlation coefficients for the comparisons of expression in angiosperm leaves and in conifer needles were still high (0.72–0.79) but were considerably lower than those for the comparisons for the different conifer species (Table 2). The values for the correlation coefficients were little affected by the method of normalization employed (data not shown).

Table 2. Pairwise comparisons of gene expression. Pearson correlations ($r_p$) on log transformed data, Spearman correlations ($r_s$) with 95% confidence intervals, and squares of Spearman correlations ($r_s^2$) of expression values (mean of three replicates) for the 384 genes on the array, pairwise for species or cell type. Data were normalized according to method 1 (based on equalization of means for the total expression of all the genes in the array for each replicate, see Materials and methods); then means were calculated for each gene over the three replicates. Pairwise Spearman correlations (and SE) for replicates within a species were for needles 0.71 (0.02) and for embryogenic callus 0.73 (0.04)

| Materials | $r_p$ | $r_s$ | $r_s^2$ |
|---|---|---|---|
| Needles | | | |
| *taeda:sylvestris* | 0.931 | 0.928 ± 0.016 | 0.861 |
| *taeda:abies* | 0.901 | 0.881 ± 0.025 | 0.776 |
| *sylvestris:abies* | 0.916 | 0.905 ± 0.021 | 0.820 |
| Embryogenic callus | | | |
| *taeda:sylvestris* | 0.845 | 0.834 ± 0.034 | 0.696 |
| *taeda:abies* | 0.835 | 0.826 ± 0.036 | 0.682 |
| *sylvestris:abies* | 0.912 | 0.912 ± 0.019 | 0.832 |
| Embryogenic callus; needles | | | |
| *taeda* | 0.870 | 0.853 ± 0.031 | 0.728 |
| *sylvestris* | 0.782 | 0.761 ± 0.047 | 0.579 |
| *abies* | 0.707 | 0.680 ± 0.060 | 0.463 |
| *N. tabacum* leaf; conifer needles | | | |
| *tabacum:taeda* | 0.763 | 0.725 ± 0.053 | 0.526 |
| *tabacum:sylvestris* | 0.745 | 0.720 ± 0.054 | 0.518 |
| *tabacum:abies* | 0.825 | 0.792 ± 0.042 | 0.628 |

### Differences in gene expression between needles and embryogenic callus

The aim here was to find if heterologous probes could be used to identify tissue-specific gene expression over species boundaries. Genes differentially expressed between embryogenic callus and needles across the three conifer species were investigated. We also examined the sensitivity of the statistical tests to the method used for normalizing the data among replicates both within and between cell-types and species, and the level of agreement among alternative methods of statistical testing.

For this comparison, data from all three species were combined (i.e. nine arrays from embryogenic callus were compared with nine arrays from needles). First the ratio of the mean expression of each gene (relative to the mean expression of all genes in the array) in embryogenic callus to that in needles was calculated. The results after normalization by method 1 and method 4 were quite

Copyright © 2002 John Wiley & Sons, Ltd. 

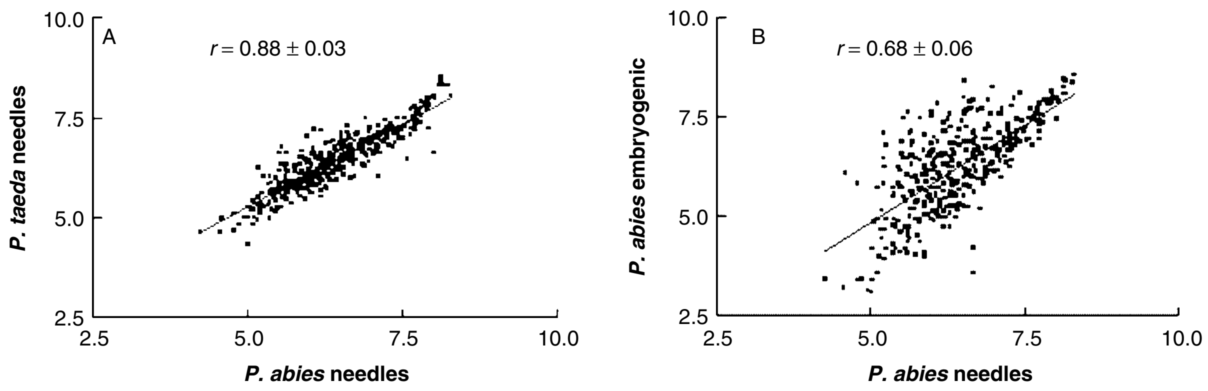

**Figure 3.** The correlation on log transformed data of estimated gene expression in needles of *Pinus taeda* and *Picea abies* (A) or embryogenic callus and needles of *Picea abies* (B). Each point represents the mean over three randomized replicates of the expression of each of the 384 genes represented in the *Pinus taeda* array, estimated by hybridization of the array with labelled cDNA from the material indicated; $r =$ Spearman correlation coefficient with 95% confidence limits similar (Table 3). Of the 30 genes, most downregulated in embryogenic callus, the first seven ranked the same by both methods, and 25 were common to both lists. Of the 30 genes most upregulated in embryogenic callus, only the most upregulated gene ranks the same in both lists, but 29 genes are common to both lists. In short, the method of normalization does not affect the classification of genes as up- or downregulated but affects the precise ordering of the genes. Then the significance of the difference in mean expression was calculated by direct $t$-testing or by the permutation method. Genes showing significant ($p < 0.01$) two-fold changes across cell-type by the direct $t$-test are listed in Table 4 with $p$ values estimated after four methods of normalization. The total number of genes showing significant ($p < 0.01$) up- or downregulation from embryogenic callus to needles across the three conifer species, by the direct $t$-test assuming unequal variances or by permutation analysis, were also compared (Table 5).

The genes showing significant two-fold changes are the same by direct $t$-tests and by permutation analysis (Table 4) but the total number of genes showing significant changes, i.e. including changes smaller than two-fold, is 33–67% greater by the permutation analysis (Table 5). The overall correlation between the two methods for all genes was high (e.g. $r = 0.9986$ after normalization by method 3b). Significance levels are higher after Z-normalization than after normalization by the other two methods, both by the direct $t$-test and by the permutation analysis (Table 4).

### Discussion

### Degree of sequence identity required for hybridization to genes in the arrays at various washing stringencies

Our results show that labelled cDNA from the three conifer species hybridized with the *Pinus taeda* arrays with comparable efficiency (Table 2). This is to be expected in view of the high sequence similarity for those genes that have been sequenced in both *Pinus* and *Picea*, usually 90% or higher nucleotide identity (Table 6). Most of the genes on the array were chosen because they were functionally identifiable from amino acid similarity to known genes, usually angiosperm genes. To that extent the genes in the array are biased toward conservation. To assess the effect of this possible bias, correlations were recalculated for the subset of 86 unidentified genes. For needles, the Spearman correlations were essentially unchanged, in fact slightly higher (see Results). For embryogenic cultures, the correlations were lower for the subset than for the complete set of 383 genes, particularly for pairwise comparisons across genera. This probably reflects the reduced constraints on regular growth in tissue culture, i.e. in part reflects chance differences in the degree to which genes are being expressed, rather than differences in the *Pinus* and *Picea* genomes.

How similar does a labelled cDNA have to be with the DNA of the target spot in an array for detectable levels of hybridization? This is usually considered for cross-reactions on microarrays

Copyright © 2002 John Wiley & Sons, Ltd.

Table 3. Comparison of the ratio of gene expression in embryogenic callus to that in needles (E : N) averaged over the three conifer species (three replicates per species) for two methods of normalization, by mean (method 1) or by slope of the regression line (method 4). For details of the two methods, see Materials and methods. The ratios E : N are sorted in ascending order (columns 2–5) or descending order (columns 6–9) of the first 30 genes. 'Gene nr' identifies the gene in the arrays. Both the size of the gene expression ratio and its position in the ordered sequence change somewhat according to the method of normalization. **Bold** genes have the same position after both normalization methods, roman genes change position and *italic* genes appear only in one column

| | Method 1 | | Method 4 | | Method 1 | | Method 4 | |
|---|---|---|---|---|---|---|---|---|
| | E : N | Gene nr | E : N | Gene nr | E : N | Gene nr | E : N | Gene nr |
| 1 | 0.217 | **256** | 0.254 | **256** | 2.907 | **136** | 2.825 | **136** |
| 2 | 0.253 | **268** | 0.297 | **268** | 2.278 | 169 | 2.572 | 78 |
| 3 | 0.307 | **273** | 0.334 | **273** | 2.215 | 179 | 2.307 | 363 |
| 4 | 0.379 | **252** | 0.415 | **252** | 2.201 | 34 | 2.273 | 169 |
| 5 | 0.514 | **250** | 0.517 | **250** | 2.160 | 78 | 2.132 | 179 |
| 6 | 0.522 | **67** | 0.539 | **67** | 2.077 | 257 | 2.118 | 34 |
| 7 | 0.565 | **306** | 0.575 | **306** | 2.072 | 104 | 2.067 | 69 |
| 8 | 0.576 | 73 | 0.593 | 187 | 2.066 | 69 | 2.055 | 76 |
| 9 | 0.576 | 325 | 0.617 | 259 | 2.046 | 363 | 2.052 | 298 |
| 10 | 0.585 | 255 | 0.618 | 73 | 2.012 | 298 | 2.044 | 104 |
| 11 | 0.591 | 187 | 0.623 | 325 | 2.006 | 76 | 2.016 | 358 |
| 12 | 0.617 | 259 | 0.623 | 255 | 1.999 | 20 | 2.014 | 257 |
| 13 | 0.621 | 348 | 0.645 | 210 | 1.892 | 297 | 1.921 | 32 |
| 14 | 0.622 | 210 | 0.656 | 59 | 1.881 | 32 | 1.910 | 20 |
| 15 | 0.630 | 145 | 0.658 | 232 | 1.879 | 91 | 1.907 | 297 |
| 16 | 0.633 | 232 | 0.659 | 348 | 1.854 | 195 | 1.891 | 217 |
| 17 | 0.633 | 191 | 0.659 | 261 | 1.853 | 217 | 1.887 | 160 |
| 18 | 0.637 | 103 | 0.663 | *44* | 1.795 | 204 | 1.863 | 91 |
| 19 | 0.643 | 243 | 0.671 | 145 | 1.791 | 160 | 1.853 | 105 |
| 20 | 0.651 | *146* | 0.683 | 272 | 1.787 | 300 | 1.830 | 195 |
| 21 | 0.658 | 227 | 0.687 | 52 | 1.777 | 108 | 1.821 | 300 |
| 22 | 0.659 | 261 | 0.689 | 227 | 1.758 | 105 | 1.791 | 108 |
| 23 | 0.664 | 272 | 0.692 | 38 | 1.756 | 135 | 1.743 | 204 |
| 24 | 0.668 | 59 | 0.692 | 103 | 1.718 | 358 | 1.740 | 269 |
| 25 | 0.671 | 52 | 0.697 | 243 | 1.698 | 320 | 1.727 | 320 |
| 26 | 0.673 | *112* | 0.698 | 191 | 1.651 | 181 | 1.701 | 135 |
| 27 | 0.688 | 38 | 0.699 | *149* | 1.626 | 296 | 1.696 | 296 |
| 28 | 0.692 | *318* | 0.705 | *323* | 1.582 | 269 | 1.632 | 181 |
| 29 | 0.699 | *107* | 0.707 | *173* | 1.570 | 361 | 1.593 | 361 |
| 30 | 0.700 | *22* | 0.714 | *205* | 1.563 | *237* | 1.588 | 374 |

among genes that are different members (paralogues) of the same family, since such cross-reactions may seriously affect interpretation of the data. Here the question concerns cross-hybridization of the array with labelled cDNA from another species; how closely related must the species be for meaningful results? Recent estimates are that considerable cross-hybridizations, even after standard high stringency washing, occur when the labelled cDNA shows more than 70% sequence identity over a length greater than 200 bp (Richmond *et al.*, 1999; Richmond and Somerville, 2000). A single short region of 70–90% identity caused little hybridization, but shorter regions of identity spread over the length of the target resulted in significant hybridization (Heller *et al.*, 1997). Table 6 shows similarities for some of the *Pinus taeda* genes in the present array. Only two of the five genes included in the table are regarded as highly conserved, but the *P. taeda* cDNA on the arrays from all of them, as expected, cross-hybridized with cDNA, not just from the conifers, but also from the angiosperm, tobacco (data not shown). This degree of sequence identity is probably sufficient to explain the quite high Spearman correlation (0.72–0.79) of gene expression in tobacco leaves with that in conifer needles (Table 2) as measured in each case by hybridization with the conifer array. The degree of cross-reaction was reduced somewhat by increasing the stringency of the final wash from 0.5×

Copyright © 2002 John Wiley & Sons, Ltd. 

Table 4. Genes, identified by gi genebank number (or sequencing project designation) and gene number in the array, showing significant two-fold up- or downregulation from embryogenic callus to needles across the three conifer species. The ratio of mean expression in embryogenic callus to that in needles after normalization by mean (method 1) is denoted E:N. Array results were normalized by method 3a (Z-score), method 3b (log transformation followed by Z-score normalization), method 4 (regression) or method 1 (mean). The significance of differences for each gene in mean values for embryogenic callus and needles was estimated by direct *t*-test, assuming unequal variances (after log transformation except for method 3b) or permutation and *P*-values are entered in the last four columns, after direct *t*-test above, (after permutation below in parenthesis)

| | | | Normalization method | | | |
|---|---|---|---|---|---|---|
| **Gene description** | **Gene nr in array** | **E:N** | **Z** | **log, Z** | **regression** | **mean** |
| Oxygen-evolving enhancer protein, 5859844 | 256 | 0.22 | 0.00000673<br>(<E-05 | 0.00000729<br><E-05 | 0.004132<br>2.06E-05 | 0.003447<br><E-05) |
| Sucrose phosphate synthase, 5903922 | 268 | 0.25 | 1.06E-07<br>(<E-05 | 1.12E-08<br><E-05 | 0.000195<br>2.06E-05 | 5.62E-06<br><E-05) |
| Water-stress inducible protein, 5858873 | 273 | 0.31 | 0.00000348<br>(<E-05 | 0.00000238<br>E-05 | 0.000302<br>0.000123 | 5.34E-06<br><E-05) |
| Ferredoxin precursor, 5859560 | 252 | 0.38 | 0.00154<br>(0.000185 | 0.001488<br>2.06E-05 | 0.001468<br>0.00496 | 0.000322<br>0.000144) |
| S-adenosylmethionine decarboxylase, 3365555 | 298 | 2.01 | 0.00000725<br>(<E-05 | 0.00000524<br><E-05 | 0.00278<br>0.000946 | 7.85E-07<br><E-05) |
| Elongation factor 1$\alpha$, 5649823 | 76 | 2.01 | 0.003987<br>(0.00372 | 0.009265<br>0.00798 | 0.178411<br>0.0262 | 0.05651<br>0.0103) |
| Chaperonin protein, 3366063 | 363 | 2.05 | 0.006565<br>(0.00483 | 0.00552<br>0.0043 | 0.088966<br>0.0383 | 0.036007<br>0.0209) |
| High molecular weight heat shock protein, 3365840 | 69 | 2.07 | 0.000231<br>(0.000247 | 0.00027<br>0.000165 | 0.006888<br>0.00413 | 0.00063<br>0.000329) |
| L-ascorbate peroxidase, 3365736 | 104 | 2.07 | 0.00000246<br>(<E-05 | 8.92E-07<br><E-05 | 0.001978<br>0.00202 | 3.92E-06<br><E0.05) |
| Unknown protein, 5649824 | 78 | 2.16 | 0.002308<br>(0.000123 | 0.026966<br><E0.05 | 0.04893<br>0.0121 | 0.015241<br>0.00245) |
| Unknown protein, 18H12 | 34 | 2.20 | 5.26E-08<br>(<E-05 | 0.00000544<br><E-05 | 0.00000337<br>0.00202 | 6.42E-06<br><E-05) |
| Glyceraldehyde-3-phosphate DH, N55F8 | 179 | 2.22 | 0.0000705<br>(<E-05 | 0.00000863<br><E-05 | 0.00305<br>0.00257 | 5.03E-05<br><E-05) |
| Unknown protein, 6696889 | 169 | 2.28 | 0.00000276<br>(<E-05 | 0.00000095<br><E-05 | 0.000893<br>0.00103 | 4.46E-07<br><E-05) |
| 25S rRNA, 6696091 | 136 | 2.90 | 9.32E-07<br>(<E-05 | 0.00000408<br><E-05 | 0.0000439<br>8.23E-05 | 3.56E-06<br><E-05) |

SSC to 0.1× SSC at 60 °C (data not shown), but the standard washing stringency was comparable with that followed by other workers using fluorescent labels. Hybridizing *Arabidopsis* cDNA with similar *Pinus taeda* arrays on glass confirmed the high degree of cross-reaction (data not shown) between angiosperms and gymnosperms. cDNA microarrays often do not distinguish closely related members of gene families. Results reflect the response of the most abundant members. To prevent cross-reaction among members of a gene family, either the cDNAs used to print the arrays need to be restricted to non-conserved regions, or the labelled probe cDNA will have to be shortened to the 3′-prime ends (Hertzberg *et al.*, 2001a). While four of the putative negative control genes

Copyright © 2002 John Wiley & Sons, Ltd. 

Table 5. Numbers of genes showing significant ($p < 0.01$, $p < 0.001$) up- or downregulation from embryogenic callus to needles across the three conifer species, by the direct *t*-test assuming unequal variances or by permutation analysis. Data were normalized by the same methods as in Table 4

| Normalization method | Z | log, Z | Regression | Mean |
|---|---|---|---|---|
| Direct *t*-test, $p < 0.01$ | 54 | 43 | 17 | 38 |
| Permutation test, $p < 0.01$ | 73 | 72 | 28 | 62 |
| Direct *t*-test, $p < 0.001$ | 23 | 22 | 4 | 19 |
| Permutation test, $p < 0.001$ | 33 | 42 | 11 | 30 |

showed only a background level of expression equal to that of the open control, the other six, particularly *gusA*, which appears unsuitable as a negative control, showed detectable cross-reaction (see Results). The gene *gusA* shows sequence similarity with other genes in the databanks.

The number of genes distinguishing pine and spruce is probably small. Humans and chimpanzees differ strikingly in anatomy and behaviour, yet the coding regions of genes studied to date are 98–99% identical, and only one human gene is known to be absent in chimpanzees (Gibbons, 1998; Gagneux and Varki, 2001).

### Use of fully randomized replicates

In much early work with microarrays, the replicate spots were printed side-by-side, thus introducing bias. This problem has been avoided here by using fully randomized replicates. The analysis can be performed essentially by regarding the replicates on membranes as blocks in a field trial and applying standard agricultural statistics (Kerr *et al.*, 2000). Lee *et al.* (2000) considered that at least three replicates were required to distinguish expressed from non-expressed genes in their experiments.

The sensitivity of the procedures described was tested by estimating the statistical significance of small changes in gene expression between embryogenic callus and needles (Tables 4, 5). Here results for the three species were pooled, so that nine arrays were compared for expression in each cell-type. After Z-normalization on log-transformed

Table 6. Sequence identities with some *Pinus taeda* genes in the array, of corresponding genes in related conifers and angiosperms, estimated by BLAST analysis

| Gene (*Pinus taeda*) | Other species | Length of sequence (bp) | % identity |
|---|---|---|---|
| Actin | *Picea rubens* (6103622) | 491 | 95 |
| (5423924) | *Cycas revoluta* (2253215) | 392 | 85 |
| | *Arabidopsis thaliana* (1949306) | 176 | 86 |
| | *Nicotiana tabacum* (1498347) | 392 | 78 |
| | *Solanum tuberosum* (21523) | 229 | 85 |
| Porin Mip1 | *Picea abies* (2258134) | 360 | 88 |
| (5423925) | *Vitis vinifera* (8886719) | 233 | 82 |
| | *Zea mays* (4768910) | 273 | 80 |
| | *Nicotiana tabacum* (1458092) | 505 | 74 |
| CAD | *Pinus radiata* (1465775) | 1315 | 98 |
| (558386) | *Picea abies* (393442) | 605 | 91 |
| | *Nicotiana tabacum* (19838) | 194, 359 | 76, 71 |
| PAL | *Pinus banksiana* (2352950) | 366 | 100 |
| (1143311) | *Pinus monticola* (6455961) | 578 | 93 |
| | *Arabidopsis thaliana* (15928192) | 681 | 72 |
| | *Nicotiana tabacum* (633596) | 845 | 72 |
| Ubiquitin | *Picea abies* (12583568) | 508 | 92 |
| (5423903) | *Arabidopsis thaliana* (2073547) | 398 | 84 |
| | *Nicotiana tabacum* (8547147) | 398 | 81 |

Numbers in parenthesis are gi numbers for the genebanks. CAD, cinnamoyl alcohol dehydrogenase; PAL, phenylalanine ammonia lyase.

Copyright © 2002 John Wiley & Sons, Ltd.

data, (method 3b), 22 genes showed significant changes in expression at $p < 0.001$ by direct $t$-tests, and 43 genes at $p < 0.01$; the corresponding figures after permutation analysis were 42 and 72 (Table 5). Some of the changes of 1.6-fold in expression were significant at $p < 0.01$. When changes of at least two-fold are considered, the results are reasonably independent of the method of normalization (Table 4 and data for the permutation test not shown). The significance levels are, however, higher after Z-normalization than after normalization by the two other methods. Normalization by the regression method, unlike the other methods tested, failed to equalize the mean expression of all genes in the array among replicates, among species, or between tissues, a prerequisite for a meaningful analysis in the present study. In short, Z-normalization on log-transformed data was the most effective method of normalizing the data. Permutation tests are preferable to direct $t$-tests on theoretical grounds as 'exact methods', but the more convenient and familiar direct $t$-tests performed well if with less sensitivity.

If 384 genes are studied and the critical probability is taken as $p = 0.01$, three to four genes are expected to show significant change in expression by chance. The choice of critical probability depends on the relative undesirability of type 1 and type 2 errors in the particular circumstances, and on whether the results can be confirmed by alternative experimental methods.

Exact methods, such as permutation tests, for significance testing are practical nowadays with modern computers (Good, 1993). The full permutation analysis examining all 48 620 arrangements of the data gave results in reasonably good agreement with the conventional $t$-test (Tables 4–5). Reducing the number of permutations in the analysis from the complete series of 48 620 to 1000 leads to serious errors and cannot be recommended; after normalization by method 3b, 39 genes were wrongly classified for significance with $p = 0.01$ as the cutoff point. More genes showed significant differences in expression between embryogenic callus and needles by the complete permutation analysis than by a direct $t$-test (Tables 4, 5), but all genes selected by the direct $t$-test were also selected by the permutation method (data not shown). The permutation analysis is more sensitive in that it detects smaller changes in expression; the changes in expression less than 1.6–2-fold, however, are in general unlikely to have much biological significance, although they may be important in particular circumstances. Two of the genes showing very high significance ($p < 0.00001$) by the permutation test were not significant even at $p < 0.05$ by the direct $t$-test (data not shown).

### Differences in gene expression among species and tissues

In pairwise comparisons of gene expression for needles correlations were slightly higher for species of the same genus than for species from different genera (Table 2). Correlations were higher for needles than for embryogenic callus (Table 2), presumably because callus cultures are more variable than needles, both in cell composition and over time. Part of the explanation for the more similar gene expression (at least when the complete set of genes is considered) for callus of *P. abies* and *P. sylvestris* than for *P. taeda:P. sylvestris* or *P. taeda:Picea abies* may be that the supply of growth regulators was the same for *P. abies* and *P. sylvestris* but different for *P. taeda*.

A detailed study of changes in gene expression from embryogenic cultures to needles was inappropriate in view of the great differences in cultural conditions, but at least some of the genes related to photosynthesis were expected to be less expressed in the dark-grown embryogenic cultures than in needles. From the permutation test after Z-normalization (method 3b), of the 13 genes broadly related to photosynthesis, four (ferredoxin precursor, rubisco, oxygen-evolving enhancer protein, sucrose phosphate synthase) were upregulated highly significantly ($p < 0.001$) in needles, and CAB was upregulated significantly ($p = 0.0170$); none were downregulated significantly.

### Concluding remarks

The data presented here support the conclusion, expected from the high sequence identity of genes from related species, that arrays printed from one species of *Pinus* or *Picea* give useful information from hybridization with labelled cDNA from other species of *Pinus* or *Picea*. This will reduce the need for mass cDNA or genomic DNA sequencing projects and allow more forest tree laboratories

Copyright © 2002 John Wiley & Sons, Ltd.

to exploit the opportunities opened for studying simultaneously a large fraction of the genes expressed in a particular tissue or cell type.

### Acknowledgements

The work was supported by a grant to S. von Arnold and R. Sederoff from the Swedish Foundation for International Cooperation in Research and Higher Education, to R. Sederoff from the NSF (USA) Plant Genome Program Grant DBI, to L. Zelena from the Swedish Institute and to Y. Chen from the Chinese Scholarship Council. We warmly thank Professor Bruce Weir for his advice on permutation methods and for discussion of the manuscript, and Dr Ross Whetten and Dr Y.-H. Sun for generous advice on microarray methods. Tobacco RNA was kindly provided by Dr Malin Elfstrand.

### References

Allona I, Quinn M, Shoop E, *et al*. 1998. Analysis of xylem formation in pine by cDNA sequencing. *Proc Natl Acad. Sci USA* **95**: 9693–9698.

Bozhkov PV, von Arnold S. 1998. Polyethylene glycol promotes maturation but inhibits further development of *Picea abies* somatic embryos. *Physiol Plant* **104**: 211–224.

Cairney JC, Xu N, Pullman GS, Ciavatta VT, Johns B. 1999. Natural and somatic embryo development in loblolly pine. *Appl Biochem Biotechnol* **77**(79): 5–17.

Chang S, Puryear J, Cairney J. 1993. A simple and efficient method for isolating RNA from pine trees. *Plant Mol Biol Rep* **11**: 113–116.

Gagneux P, Varki A. 2001. Genetic differences between humans and great apes. *Mol Phylogenet Evol* **18**: 2–13.

Gibbons A. 1998. Which of our genes makes us human? *Science* **281**: 1432–1434.

Girke T, Todd J, Ruuska S, White J, Benning C, Ohlrogge J. 2000. Microarray analysis of developing *Arabidopsis* seeds. *Plant Physiol* **124**: 1570–1581.

Good P. 1993. *Permutation Tests: A Practical Guide to Resampling Methods for Testing Hypotheses*. Springer-Verlag: New York.

Gupta PK, Durzan DJ. 1986. Somatic embryogenesis from callus of mature sugar pine embryos. *Biotechnology* **4**: 643–645.

Hegde P, Qi R, Abernathy K, *et al*. 2000. A concise guide to cDNA microarray analysis. *BioTechniques* **29**: 548–562.

Heller RA, Schena M, Chai A, *et al*. 1997. Discovery and analysis of inflammatory disease-related genes using cDNA microarrays. *Proc Natl Acad Sci USA* **94**: 2150–2155.

Hertzberg M, Sievertzon M, Aspeborg H, Nilsson P, Sandberg G, Lundeberg J. 2001a. cDNA microarray analysis of small plant tissue samples using a cDNA tag target amplification protocol. *Plant J* **25**: 585–591.

Hertzberg M, Aspeborg H, Schrader J, *et al*. 2001b. A transcriptional roadmap to wood formation. *Proc Natl Acad Sci USA* **98**: 14 732–14 737.

Ingestad T. 1979. Mineral nutrient requirements of *Pinus sylvestris* and *Picea abies* seedlings. *Physiol Plant* **45**: 373–380.

Kerr MK, Martin M, Churchill GA. 2000. Analysis of variance for gene expression microarray data. *J Computat Biol* **7**: 819–837.

Lee M-LT, Kuo FC, Whitmore GA, Sklar J. 2000. Importance of replication in microarray gene expression studies: statistical methods and evidence from repetitive cDNA hybridizations. *Proc Natl Acad Sci USA* **97**: 9834–9839.

Logemann J, Schell J, Willmitzer L. 1987. Improved method for the isolation of RNA from plant tissues. *Anal Biochem* **163**: 16–20.

Maleck K, Levine A, Eulgem T, *et al*. 2000. The transcriptome of *Arabidopsis thaliana* during systemic acquired resistance. *Nature Genet* **26**: 403–410.

Richmond T, Somerville S. 2000. Chasing the dream: plant EST microarrays. *Current Opin Plant Biol* **3**: 108–116.

Richmond CS, Glasner JD, Mau R, Jin H, Blattner FR. 1999. Genome-wide expression profiling in *Escherichia coli* K-12. *Nucleic Acids Res* **19**: 3821–3835.

Ruan Y, Gilmore J, Conner T. 1998. Towards *Arabidopsis* genome analysis: monitoring expression profiles of 1400 genes using cDNA microarrays. *Plant J* **15**: 821–833.

Schena M, Shalon D, Davis RW, Brown PO. 1995. Quantitative monitoring of gene expression patterns with a complementary DNA microarray. *Science* **270**: 467–470.

Schenk PM, Kazan K, Wilson I, *et al*. 2000. Coordinated plant defense responses in *Arabidopsis* revealed by microarray analysis. *Proc Natl Acad Sci USA* **97**: 11 655–11 660.

Whetten R, Sun Y-H, Zhang Y, Sederoff R. 2001. Functional genomics and cell wall biosynthesis in loblolly pine. *Plant Mol Biol* **47**: 275–291.

Copyright © 2002 John Wiley & Sons, Ltd. 
